# Docking and Molecular Dynamics-Based Identification of Interaction between Various Beta-Amyloid Isoforms and RAGE Receptor

**DOI:** 10.3390/ijms231911816

**Published:** 2022-10-05

**Authors:** Anna P. Tolstova, Alexei A. Adzhubei, Vladimir A. Mitkevich, Irina Yu. Petrushanko, Alexander A. Makarov

**Affiliations:** Engelhardt Institute of Molecular Biology, Russian Academy of Sciences, 119991 Moscow, Russia

**Keywords:** Alzheimer’s disease, beta-amyloid, RAGE, interaction interface, blood–brain barrier, transcytosis, molecular dynamics, macromolecular docking

## Abstract

Beta-amyloid peptide (Aβ) is a ligand associated with RAGE (Advanced glycosylation end product-specific receptor). Aβ is translocated in complexes with RAGE from the blood to brain across the blood–brain barrier (BBB) by transcytosis. Aβ and its isoforms are important factors in the Alzheimer’s disease (AD) pathogenesis. However, interaction with RAGE was previously studied for Aβ but not for its isoforms. The present study has been directed at identifying the key interaction interfaces between RAGE and Aβ isoforms (Aβ_40_, Aβ_42_, phosphorylated and isomerized isoforms pS8-Aβ_42_, isoD7-Aβ_42_). Two interfaces have been identified by docking: they are represented by an extended area at the junction of RAGE domains V and C1 and a smaller area linking C1 and C2 domains. Molecular dynamics (MD) simulations have shown that all Aβ isoforms form stable and tightly bound complexes. This indicates that all Aβ isoforms potentially can be transported through the cell as part of a complex with RAGE. Modeling of RAGE interaction interfaces with Aβ indicates which chemical compounds can potentially be capable of blocking this interaction, and impair the associated pathogenic cascades. The ability of three RAGE inhibitors (RAP, FPS-ZM1 and RP-1) to disrupt the RAGE:Aβ interaction has been probed by docking and subsequently the complexes’ stability verified by MD. The RP-1 and Aβ interaction areas coincide and therefore this inhibitor is very promising for the RAGE:Aβ interaction inhibition.

## 1. Introduction

Alzheimer’s disease (AD) is characterized by an abnormal accumulation of beta-amyloid (Aβ) in the brain. It is hypothesized that initial vascular disorders, and in particular impairment of the blood–brain barrier (BBB), play an important role in Aβ accumulation and neurodegeneration [1,2,3]. The BBB controls the uptake of Aβ from plasma to the brain via the multi-ligand Advanced glycosylation end product-specific receptor (RAGE) [4,5], and the removal of the brain-derived Aβ via the LRP1 receptor [6,7]. RAGE-bound Aβ is transported to the other side of the endothelial cell membrane by transcytosis. RAGE is a transmembrane protein from the immunoglobulin family, which is present in various tissues at a low level, increasing at the sites of stress and cell damage [8,9,10]. In AD, RAGE expression markedly increases in the areas of Aβ accumulation [11]. At the same time, the level of circulating soluble form of RAGE, sRAGE (23–342), is reduced in Alzheimer’s patients [12]. The binding of RAGE ligands by sRAGE prevents their interaction with the receptor form of RAGE [13]. Binding of the protein glycation end products (AGEs) or Aβ to the membrane-bound receptor form of RAGE leads to the activation of pathogenic cascades [13]. Signaling mediated by the Aβ binding to RAGE is an important contributing factor to the development of AD [14]. The interaction of Aβ with RAGE is critical for the pathology of AD and is a promising target for the development of therapy, since disruption of this complex can be achieved with the help of therapeutic substances circulating in the bloodstream [15]. Thus, the structure of the Aβ complex with RAGE is necessary for creating compounds that can prevent this interaction.

RAGE is formed by the extracellular, transmembrane and cytosolic domains. The extracellular ligand-binding RAGE domain is subdivided into three regions: a variable V domain (23–116) and two conserved domains: C1 (124–221) and C2 (227–317) [13]. The variable V domain consists of two β-sheets connected by an SS bridge. Hydrogen and hydrophobic bonds between the V and C1 domains link these domains to form a single structural unit. The molecular surface of VC1 domains contains hydrophobic cavities and positively charged regions [16,17,18,19]. Using specific antibodies, it was shown that Aβ oligomers interact with the V domain, and that Aβ aggregates interact with the C1 domain [4,20,21]. Unlike oligomers and aggregates, the effect of fibrils on cells does not change when the antibodies blocking RAGE domains are used [20]. Analysis of the crystal structure of ectodomains [22] and NMR spectroscopy of the ligand-binding domain [23] showed the existence of a hydrophobic cavity in the V domain of RAGE, which has a flexible region 55–71. This flexible region provides plasticity within the hydrophobic cavity, thereby allowing RAGE to effectively interact with ligands [22,23]. The use of peptides RAGE 60–76 [24], as well as shorter peptides 60–70 and 60–65 [25,26], reduces the negative effect of RAGE-mediated Aβ on neuronal cells due to the competitive binding of Aβ. This means that region 55–71 may well be a part of the RAGE:Aβ interaction interface.

There are Aβ isoforms in the body, which differ in the number of amino acid residues and in their modifications. One of the methods for diagnosing AD is based on the assessment of the ratio of Aβ_40_ and Aβ_42_ concentrations [8,9,27] in blood plasma. According to experimental data, the binding constant values of Aβ_40_ and Aβ_42_ are close. For example, RAGE expressed in Chinese hamster ovary cells binds Aβ_40_ with a constant of 75 ± 5 nM [1], and the binding constant of Aβ_42_ to RAGE on human neuroblastoma SH-SY-5Y cells is 92 ± 40 nM [28]. RAGE binds soluble Aβ in the V-domain region with a constant of about (Kd = 52.2 ± 14.6 nM) [29]. However, it is possible that the binding of Aβ_40_ and Aβ_42_ may differ, since the constants were not evaluated under the same conditions. Moreover, uptake of Aβ_40_ and Aβ_42_ by endothelial cells differs on the BBB luminal side (from the blood to the brain), and does not differ on the abluminal side [30]. The intrinsic fluorescence of RAGE tryptophans was found to be sensitive to Aβ binding (residues 54, 61, 72). A study of the binding of truncated Aβ variants to RAGE showed that the main region of Aβ that binds the V domain is 17–23. This region contains strongly hydrophobic residues 17-LVFFA-21, flanked by the negatively charged 22-DE-23 residues at the C-terminus [15]. The Aβ_40_ binding constant with V-RAGE is 1.6 × 10^6^ M^−1^ (Kd = 0.6 μM), which is lower than for full-length RAGE. Accordingly, the Aβ_16–23_ peptide effectively blocks the RAGE-dependent uptake of Aβ_40_ by mouse BBB endothelial cells [15]. These data do not correlate well with the current model of the dimeric RAGE complex binding the Aβ dimer, which predicts that the three negatively charged Aβ residues (E3, D7, E11) are the most important for binding [31].

To summarize, Aβ forms stable complexes with RAGE with a high binding constant. Aβ predominantly interacts with the V and C1 domains, however the exact interface is as yet unknown.

Currently, the development of therapy aimed at blocking the binding of Aβ to RAGE offers a promising avenue [29]. In particular, the RP-1 peptide, which binds to RAGE with high affinity and has a high homology with region 16–23 (KLVFFAED) of the Aβ peptide, is undergoing preclinical trials [32]. It was shown on the SH-SYSY cell culture that RP-1, by binding to RAGE, prevents Aβ-induced cellular stress [33]. The RAGE antagonistic peptide (RAP) that blocks the RAGE signaling pathway was designed based on the RAGE-binding domain of the HMGB1 protein and was shown to be effective in acute lung inflammation [34]. It can inhibit Aβ peptide binding to RAGE [35]. The other promising therapeutic agent to treat AD patients is FPS-ZM1, a high-affinity RAGE-specific blocker, that can inhibit Aβ binding to RAGE [36]. It was shown that FPS-ZM1 can reduce neurological damage and inflammation in the APP(sw/0) transgenic mouse model of AD [36] and prevent neuronal death induced by astrocytes over-expressing the ALS-linked mutant hSOD1G93A [35].

It has now become clear that the seeds of pathological aggregation, representing the chemically and/or structurally altered Aβ molecules [37], that induce the transition of endogenous Aβ molecules from the normal monomeric state to neurotoxic oligomers and amyloid plaques [38]. The Aβ isoform with an isomerized Asp7 residue (isoD7-Aβ_42_) acts as a seed of cerebral amyloidogenesis [39]. In addition, isomerization leads to an increase in Aβ_42_ cytotoxicity [40]. However, if the Ser8 residue is phosphorylated, then the amyloidogenic effects of isoD7-Aβ_42_ are neutralized [41]. Phosphorylated Aβ reduces zinc-dependent oligomerization and the amount of amyloid plaques in the brain of animals with AD [42]. Changes in the pattern of transport of modified Aβ_42_ isoforms through the BBB from blood to brain, which is mediated by RAGE, may affect the severity of Alzheimer’s disease. Thus, it is important not only to study the Aβ_42_ interaction with the RAGE interface, but also to determine how exactly these modifications of Aβ affect the nature of their interaction with RAGE.

In view of such differing effects of Aβ_42_ isoforms, the question of the interaction interface between various isoforms of Aβ and RAGE is very important. In this study, a strong but non-specific binding of Aβ_40_ and Aβ_42_ to RAGE has been shown by molecular docking and MD modeling. Post-translational modification of Aβ_42_ to isomerized iso-D7-Aβ_42_ and phosphorylated pS8-Aβ_42_ did not change dramatically the interaction interfaces of RAGE with Aβ_42_ isoforms, however iso-D7-Aβ_42_ and pS8-Aβ_42_ have shown more specific interactions compared to native Aβ_42_ isoform. Thus, we can anticipate that all isoforms can freely penetrate the BBB via complex formation with RAGE. The three RAGE inhibitors that display an ability to hinder the RAGE:Aβ interaction have been studied. RAGE:RAP and RAGE:FPS-ZM1 interaction interfaces represent compact localized areas within the VC1 domain of the RAGE protein. Therefore, one can conclude that these inhibitors cannot block the whole region of potential Aβ interaction on the RAGE surface. The RP-1 and Aβ interaction areas coincide and therefore this inhibitor is very promising for the RAGE:Aβ interaction inhibition.

## 2. Results

To the best of our knowledge, the RAGE structure was not fully resolved experimentally, since only the structure of the first two domains was available. However, the entire protein structure is currently available from the AlphaFold database [43], modeled by a powerful program for predicting the structure of proteins using neural networks. This structure was obtained, placed into a membrane according to the UNIPROT data, and subjected to a 50 ns MD production run to acquire an equilibrium RAGE conformation in an aqueous saline solution (pH 7.0). The domain’s structure remained stable after MD with the membrane located at a substantial distance from the domains (Figure S1). Therefore, we used a part of the RAGE structure containing residues 23–330 (structure lacking the signal peptide, C-terminal disordered tail and the membrane segment of the protein) in order to save computational resources.

### 2.1. Docking and MD Modeling of Aβ Isoforms to RAGE

Utilizing this structure, a global docking of the Aβ_42_ peptide using numerous protein–protein docking servers was performed. The particular interaction interface between RAGE and Aβ depended on the docking algorithm and varied significantly for the average complexes from different servers. The choice of a large set of docking software was justified by the increased reliability of overall docking results obtained by combining diverse software.

The docking results showed a certain selectivity in the interaction of RAGE with Aβ_42_ (Figure S2), however, the Aβ_42_ molecule could be docked almost everywhere on the RAGE surface. We selected as binding sites all of the RAGE regions where the number of contacts with Aβ_42_ for each RAGE residue was greater than 600. These regions are shown in Figure S2B.

Figure S2B shows that the interaction area can be structurally divided into two regions. Interface 1 includes residues 25–35, 57, 73, 77, 116–118, 123, 150, 186, 216–221 and is located between the V domain and C1 domain, Interface 2 includes the residues 198, 230, 233, 237, 314 and is located between domains C1 and C2.

There is evidence that Aβ_40_ and Aβ_42_ each interact with RAGE in a different manner, resulting in a different rate and effectiveness of penetration through the BBB [1,28]. We performed global docking of the Aβ_40_ peptide to RAGE using the same set of docking servers as for Aβ_42_. The results are shown in Figure S3.

**Figure 1:**
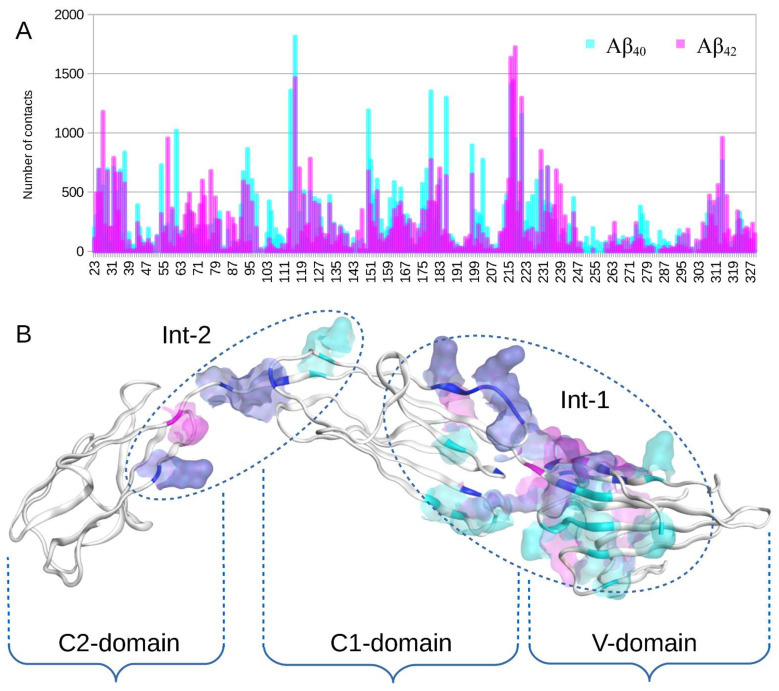
Global docking results of Aβ_42_ and Aβ_40_ peptides to RAGE protein performed with servers LzerD, ATTRACT, PatchDock, ClusPro, Hdock and Zdock and analyzed with QASDOM server. The 30 best complexes from each server have been used for analysis, and 10 complexes from Zdock. (**A**) Superposition of the docking contact frequency histogram for RAGE residues over all 160 complexes with Aβ_42_ (cyan) and Aβ_40_ (magenta). (**B**) Interacting residues highlighted in the RAGE structure according to the global docking results. Residues interacting only with Aβ_42_ are highlighted with magenta. Residues interacting only with Aβ_40_ are highlighted with cyan. Residues interacting with both isoforms are highlighted with blue. RAGE domains are indicated with curly braces. In (**B**), two independent extended interaction regions localized far from each other can be distinguished. They are marked with dotted ovals and represent Interface 1 (Int-1) and Interface 2 (Int-2).

Comparison of global docking results for Aβ_40_ and Aβ_42_ showed that Aβ_40_ formed slightly more contacts spread over a greater RAGE surface compared to Aβ_42_ (Figure 1, Figures S2 and S3). However, overall, the interaction surfaces were almost the same (Figure 1).

To study the effect of Aβ_42_ post-translational modifications on the Aβ_42_ BBB penetration process and interaction with RAGE we performed targeted flexible docking of the three Aβ_42_ isoforms (Aβ_42_, isoD7-Aβ_42_, pS8-Aβ_42_) using PatchDock to the two interaction interfaces in RAGE revealed by global docking.

**Figure 2:**
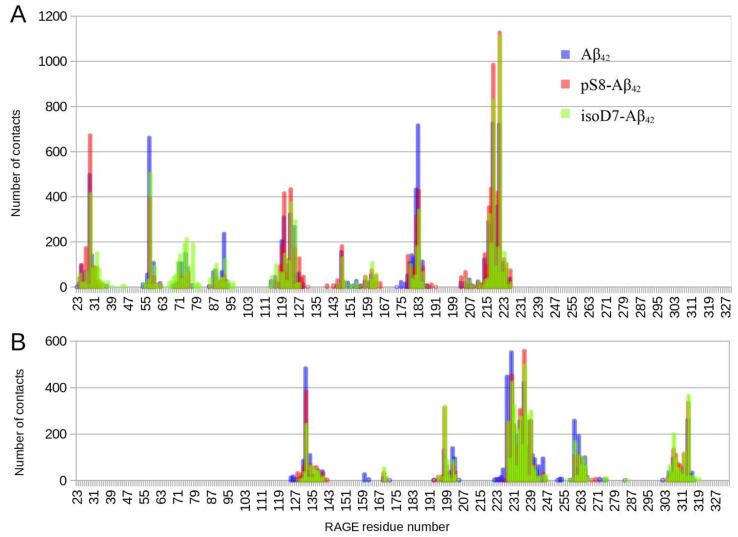
Contact frequency histogram for targeted docking of the Aβ_42_ isoforms to the previously obtained interaction area of the RAGE protein performed with PatchDock server and analyzed by QASDOM software. The 30 best complexes with each isoform have been used for the analysis. (**A**) results for targeted docking to Interface 1, (**B**) results for targeted docking to Interface 2. Contacts with Aβ_42_ are colored blue, contacts with pS8-Aβ_42_ are colored red and contacts with isoD7-Aβ_42_ are colored green.

Figure 2 shows that the interaction interfaces for the three Aβ_42_ isoforms are almost identical. Only the sizes of the peaks differ. The total number of contacts in the graph areas was about 15,000 for each isoform (15,666 for Aβ_42_, 15,217 for pS8-Aβ_42_ and 14,766 for isoD7-Aβ_42_). The relative peak maxima changed significantly for different isoforms with an almost equal number of contacts.

**Figure 3:**
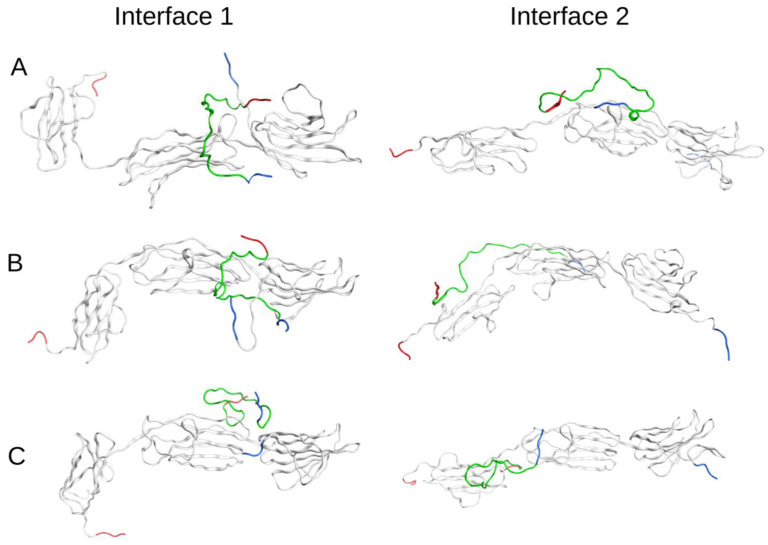
The best docking RAGE:Aβ complexes for the three Aβ_42_ isoforms after 100 ns of MD. (**A**) RAGE with Aβ_42_, (**B**) RAGE with isoD7-Aβ_42_, (**C**) RAGE with pS8-Aβ_42_. The Aβ_42_ peptides are colored green, N-termini are highlighted with blue and C-termini are highlighted with red.

The best Aβ_42_:RAGE complex models with Interface 1 for each Aβ_42_ isoform were submitted to MD simulation for 100 ns (Figure 3). They represent different parts of the full Interface 1 area in the interaction. According to the results of the MD modeling, all six selected complexes remained stable. Thus, docking shows that the interactions between RAGE and Aβ_42_ isoforms are distributed over large areas of interfaces 1 and 2 and MD modeling confirms the stable binding of all three Aβ_42_ isoforms.

We also performed global blind-docking of an additional Aβ_42_ peptide to the RAGE:Aβ_42_ complex to see if the presence of an already bound peptide changed the binding specificity. The result was negative. As before, the new peptides were bound around the protein approximately evenly without any pronounced preferences (Figure S4).

### 2.2. MD Modeling of RAGE:Aβ Interactions under Various pH

**Figure 4:**
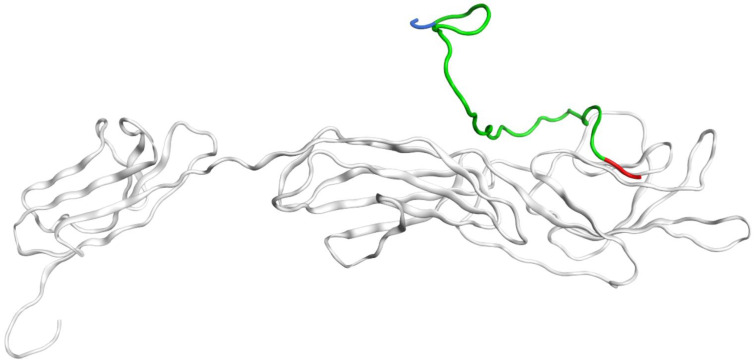
The resulting RAGE:Aβ_42_ complex after 250 ns of MD at 5.5 pH. Aβ_42_ is colored green, N-terminus is colored blue and C-terminus, red. The initial structure is shown in Figure 3A.

Beta-amyloid bound with RAGE is transported through the BBB by transcytosis [5]. After binding to the receptor, this complex is internalized and enters the common endosomal sorting network [44]. It is known that in mature endosomes, the pH ranges from 5 to 5.5 and the ion concentration is low [45]. Accordingly, it was decided to conduct MD of the best RAGE:Aβ_42_ docking complex at conditions close to endosomal to study the complex behavior in early and late endosomes. As an outcome of a 250 ns MD of RAGE:Aβ_42_ complex at pH 5.5 indicates, Aβ_42_ was interacting with RAGE throughout the simulation. The N-terminus at the middle of the simulation time started to expose into the solution (breaking contacts with RAGE), while the C-terminus continuously remained in contact with RAGE bound by a large number of hydrogen bonds (Figure 4). Therefore, in endosomal conditions, Aβ_42_ does not detach from RAGE, indicating that after transferring through the BBB, Aβ_42_ remains in complex with RAGE at least for some time.

The same results were obtained with pH 6.0. The RAGE:Aβ_42_ complex was stable during 100 ns MD simulation (Figure S5A). The RAGE:isoD7-Aβ_42_ and RAGE:pS8-Aβ_42_ complexes also were stable at pH 5.5 after 100 ns (Figure S5B,C).

### 2.3. Free Energy of Binding Calculations for RAGE:Aβ Isoforms

To study the strength of intermolecular interactions in the complexes and obtain the free energy of binding for the RAGE:Aβ_42_, RAGE:isoD7-Aβ_42_ and RAGE:pS8-Aβ_42_ complexes, umbrella sampling was performed. The simulation time of 10 ns is sufficient for the free energy of binding calculation, as in this interval the maximum of the free energy value was reached, and the potential of mean force (PMF) stopped rising (See Materials and Methods). The PMF curves and energy distribution over each window are shown in Figures S6–S8. The free energy of binding calculated from these curves for the RAGE:Aβ_42_ complex corresponds to ∆G = −17.5 ± 0.8 kcal/mol, for the RAGE:pS8-Aβ_42_ complex it corresponds to ∆G = −14.9 ± 0.7 kcal/mol and for the RAGE:isoD7-Aβ_42_ complex it corresponds to ∆G = −14.5 ± 0.6 kcal/ mol. This may indicate a higher binding constant of Aβ_42_ to RAGE compared to the modified isoforms pS8-Aβ_42_ and isoD7-Aβ_42_.

### 2.4. Docking and MD Modeling of RAP, FPS-ZM1 and RP-1 Inhibitors Interaction with RAGE

In the last few years, development of the therapy aimed at blocking the binding of Aβ to RAGE was pursued in several studies [34,36,46]. The known RAGE inhibitors were viewed as promising candidates for the RAGE:Aβ interaction inhibition. We have used three RAGE inhibitors that are also potentially able to inhibit its interaction with Aβ_42_. These are a RAP peptide [34], the compound FPS-ZM1 [36] and RP-1 peptide [46].

Global docking was performed to identify the interaction sites for RAP and RP-1 inhibitors. Subsequently, targeted docking to these sites was also been conducted. Following this, the best-rated complex for each inhibitor was submitted to MD simulation for 100 ns. Docking showed a clear and specific interaction interface between RAGE and RAP. RAP docked primarily to residues 116–125 and 179–183, in one of the RAGE interaction areas with Aβ_42_ (Figure S9). In contrast, the RP-1 docking sites were spread over the RAGE surface, resembling the contact frequency histogram for interaction with Aβ_42_ (Figure S10). This signifies that PR-1 can potentially block all of the RAGE interaction areas with Aβ_42_. In the majority of docking complexes, the RAGE protein was identified as interacting with FPS-ZM1 via residues 54, 96–98 and 114–120 intersecting with the Aβ_42_ interaction areas (Figure S11). However, there was no full overlap.

The MD modeling results for the best RAGE:RAP, RAGE:RP-1 and RAGE: FPS-ZM1 complexes showed that RAP, RP-1 and FPS-ZM1 have been tightly bound to RAGE during all MD simulations (Figures S9–S11).

## 3. Discussion

Currently there is no full-size experimentally solved RAGE protein structure in the public domain, therefore only part of the structure was available for analysis [28]. We constructed for the first time a model of the complete equilibrium structure of RAGE with a membrane, in water and saline solution. As a result of the docking of Aβ_42_ and its isoforms (Aβ_40_, isoD7-Aβ_42_, pS8-Aβ_42_) to this RAGE structure, we showed that the C2 domain not studied previously can be involved in the interaction with Aβ peptides (Int-2 in Figure 1 and Figure 2). We also found a large interaction area (Int-1 in Figure 1 and Figure 2) at the region between the V and C1 domains of RAGE, partially including both domains (Figure 1 and Figure 2), which is confirmed experimentally as a primary interaction area of the V and C1 domains in RAGE binding with Aβ [4,15,20,21,24,25,26,31,47]. At the present time there is no consensus concerning the domains that play a leading role in the interaction with Aβ. For example, the work [47] showed that the Aβ peptide interacts with the 49–52 and 108–118 β-sheets located in the V domain. In contrast, other studies [4,20,21] showed that Aβ aggregates interact only with the C1 domain. According to our data, the 49–52 site does not interact with either Aβ_40_ or Aβ_42_, while the 108–118 site, on the contrary, appears in both of the interaction interfaces. It was shown [24,25,26] that the site 55–71 in the RAGE V domain can constitute the main site in the interface between Aβ peptides and RAGE. This site is also present in the interface identified by us, although in our model the interaction with these residues is more pronounced for Aβ_40_ than for Aβ_42_ (Figure 1, Figures S2 and S3). This is in line with the data from the paper [30], which indicate that the rate of transfer of Aβ_40_ and Aβ_42_ by endothelial cells from the blood to the brain differs.

According to our data, Aβ_42_ and its isoforms interact rather with the region between the V and C1 domains, partially including each of the domains (Int-1 in Figure 1 and Figure 2), and not with two separate sites in the C1 and V domains. It is noteworthy, however, that experimentally based conclusions about the involvement of individual domains were made on the basis of experiments with only the V-domain [15,31], or using other truncated variants of the protein [24,25,26]. This means that the interaction Interface 1 identified by us and localized between the domains is outside the scope of the above studies. In addition, Aβ_42_ at various oligomeric states (from monomers to large non-fibrillar aggregates) interacts with RAGE in a dissimilar way [4,15,20,21].

Our data indicate that, in the experimental search for an interaction interface, the region between the V and C domains should represent a primary target.

A number of studies showed that different Aβ_42_ isoforms have different pathogenicity. Thus, isomerized Aβ_42_ due to a greater tendency to aggregation has greater neurotoxicity than Aβ_42_ [40], accelerating plaque formation in a mouse model of Alzheimer’s disease [39]. At the same time, phosphorylation can reduce the pathogenic effect of the isomerized isoform [41,42]. The Aβ_40_ isoform is considered to be more physiological than Aβ_42_, and the Aβ_40_/Aβ_42_ ratio is used as one of the AD markers [27]. Hence, an increase in the level of modified Aβ_42_ isoforms in the brain can affect the severity of AD. Since Aβ penetrates through the BBB from the blood to the brain by binding to RAGE, differences in binding with modified forms of Aβ_42_ can lead to a preferential transfer of one or another isoform to the brain, which is one of the AD risk factors.

According to the global docking data, in the case of Aβ_42_ the main interaction occurs in a small region between the V and C1 domains and adjacent residues (Figure 1 and Figure S2). For Aβ_40_ this region is much more extended and includes the residues not involved in the interface for Aβ_42_, such as 54, 61, 114 and 177–179 (Figure 1 and Figure S3), while the 25–35 region is involved in the RAGE:Aβ_40_ interaction to a lesser extent than for RAGE:Aβ_42_.

**Figure 5:**
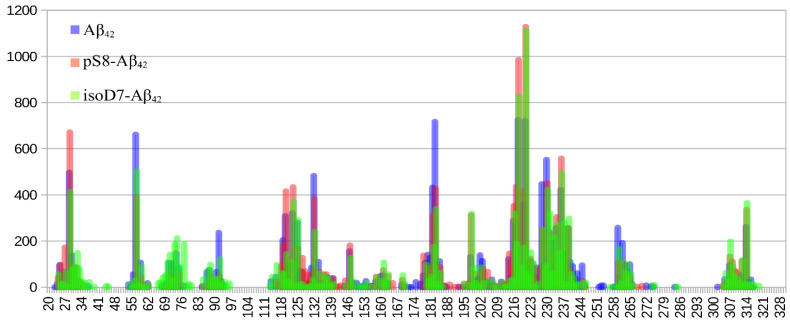
Joint contact frequency histograms for targeted docking of Aβ_42_ isoforms to Interface 1 and Interface 2 of the RAGE protein performed with PatchDock server and analyzed by QASDOM software. A total of 30 best complexes with each isoform were used for the analysis.

Targeted docking of the three Aβ_42_ isoforms to RAGE has been performed on the RAGE residues from the Interface 1 and Interface 2 identified by global docking (Figure 2). Importantly the contact histograms obtained after these docking runs overlap for each isoform. Thus, the Aβ_42_ peptides commonly interact with both Interfaces 1 and 2 concurrently. Joint histogram built using contact histograms for both interfaces showed that the maximum of contacts falls to residues 218–221 RAGE (Figure 5). From the data obtained for Aβ_42_, it is hard to differentiate a preferred region among all potential binding sites because the number of contacts for different regions is approximately the same (Figure 5). At the same time, isoD7-Aβ_42_ has a more specific RAGE binding interface than Aβ_42_, in which the 218–221 region has more than twice Aβ contacts as compared to other RAGE regions (Figure 5). For pS8-Aβ_42_, there is the same number of contacts with the 218–221 RAGE region as for isoD7-Aβ_42_. A region with a higher number of contacts appears at residues 25–30, while the peaks in the histogram of contacts at residues 55–58 and 181–183 are less pronounced compared to other isoforms.

**Figure 6:**
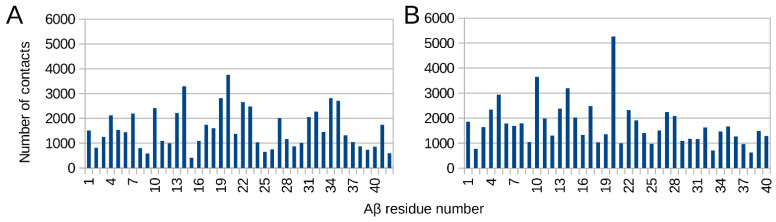
Contact frequency histogram for Aβ_42_ (**A**) and Aβ_40_ (**B**) residues over 160 complexes with RAGE. Global docking results for the Aβ_42_ and the Aβ_40_ to RAGE protein performed with servers LzerD, ATTRACT, PatchDock ClusPro, Hdock and Zdock and analyzed with QASDOM software. A total of 30 best complexes from each have been used for the analysis, and 10 complexes from Zdock.

All of the studied Aβ isoforms (Aβ_42_, Aβ_40_, pS8-Aβ_42_, isoD7-Aβ_42_) interact with RAGE over their entire surfaces. After targeted flexible docking, they wrap around RAGE, while global docking shows that the Aβ region 19–22 is characterized by a larger number of contacts compared with the other residues (Figure 6). These data are consistent with experimental data indicating that the hydrophobic region 17–23 plays an important role in the RAGE:Aβ interaction [15].

Based on the analysis of the number of contacts, it can be estimated that isoD7-Aβ_42_ displays the strongest binding to RAGE, with weaker binding for Aβ_42_, and pS8-Aβ_42_ occupying an intermediate position. Although isoD7-Aβ_42_ by our MD approximation can form more stable complexes with RAGE and, therefore, may better pass through the BBB, this isoform is also potentially more sensitive to inhibitors due to a higher selectivity of interaction compared to the native Aβ_42_.

We have verified our conclusions on the high binding constant of RAGE to Aβ_42_ and its isoforms by conducting the MD Umbrella sampling. None of the complexes have been broken during simulation either at normal pH (7.0) or at acidic pH (5.5 and 6.0) (Figure 4 and Figure S5). Correct quantitative estimates of the interaction coefficient (free energy of binding) of RAGE with Aβ_42_ are beyond modern computer capabilities, since they require a large number of resource-intensive calculations for all variants of complexes obtained from targeted docking. However, molecular dynamics allows one to estimate the approximate value of the interaction coefficient. Consequently, we completed umbrella sampling for the best-ranked docking complex of RAGE with each Aβ_42_ isoform. We hypothesize that the modeled complex formation energies will be significantly higher than those measured experimentally. Our modeling data do not take into account the entire variety of possible complex structures that can be constructed and realized within the framework of our interaction interfaces model. Until now, for the monomeric form of Aβ with post-translational modifications, the experimental binding constant to RAGE was not determined. According to the MD data, the native form of Aβ_42_ shows a strong interaction with RAGE with the binding constant of 6.95 × 10^12^ M^−1^ (free energy of binding ∆G = −17.5 ± 0.8 kcal/mol, dissociation constant K_d_ = 0.14 pM). The pS8-Aβ_42_ and isoD7-Aβ_42_ isoforms have similar constants 0.085 × 10^12^ M^−1^ and 0.043 × 10^12^ M^−1^ (free energies of binding ∆G = −14.9 ± 0.7 kcal/mol, K_d_ = 11.8 pM^-^ and ∆G = −14.5 ± 0.6 kcal/mol, respectively, K_d_ = 23.3 pM), which are lower than the constant for native Aβ_42_. An alternative way of accessing these interactions is docking which reflects the average number of contacts in contrast to MD-based calculations. Consequently, docking-based assessment can differ and indeed it shows that isoD7-Aβ_42_ displays the strongest binding to RAGE, i.e., docking showed a higher number of contacts of RAGE protein at Interface 1 with isoD7-Aβ_42_ compared to the native form of Aβ_42_. MD showed that the binding constant is higher for the native form of Aβ (for the best complex), which can also occur with a smaller number of contacts.

In order to evaluate the effectiveness of the existing RAGE inhibitors in blocking interaction with the Aβ isoforms, we have modeled and compared the interaction interfaces of some of these inhibitors, with the identified RAGE interaction interfaces of Aβ isoforms. According to our data, the RP-1 inhibitor should be the most effective as its interaction interface largely matches that for Aβ (Figure S10), and it could completely inhibit the interaction through competitive binding. The RAP inhibitor, according to the published data, can block binding of Aβ [1]. However, their interaction interface as it is described [1,34] and confirmed by us (Figure S8) does not include RAGE residues 55–71, important for the Aβ binding according to other studies [24,25,26]. The low molecular weight inhibitor FPS-ZM1 partially blocked interface 1 and did not block interface 2 at all (Figure S11). It can be estimated that its effectiveness in preventing the Aβ binding to RAGE would be low.

Since RAGE-associated ligands are translocated across the BBB by transcytosis [5], we evaluated the effect of various pH values that can prevail in vesicles, on the stability of RAGE complexes with Aβ isoforms. Endosomal sorting determines either degradation of macromolecules or transcytosis by fusion with abluminal plasma membrane [44]. Our MD results show that even at pH 5.5, the complexes remained stable, indicating that all Aβ isoforms can be transported through the cell in complex with RAGE.

## 4. Materials and Methods

### 4.1. Structure Preparation

The initial structure of the human RAGE (Advanced glycosylation end product-specific receptor) was obtained from the AlphaFold protein structure database [43]. The DDPC membrane and water with NaCl at 0.115 mM concentration were added and subsequent relaxation for 50 ns by molecular dynamics (MD) using GROMACS [48] software was performed to obtain an equilibrium RAGE conformation. The structure of the Aβ_42_ peptide was previously modeled by us [49]. The Aβ_42_ modifications with phosphorylated Ser8 (pS8-Aβ_42_) and isomerized Asp7 (isoD7-Aβ_42_) were constructed by expert modeling. Aβ_40_ structure was obtained from the PDB Bank of protein structures, entry PDB:2LFM [50]. The structure of the RAP inhibitor was constructed by expert modeling according to the PubChem entry https://pubchem.ncbi.nlm.nih.gov/compound/127021052 (accessed on 18 February 2022). The structure and topology of the FPS-ZM1 inhibitor was obtained by the ATB server [51], according to the PubChem entry https://pubchem.ncbi.nlm.nih.gov/compound/24752728 (accessed on 18 February 2022). The structure of the RP-1 peptide was constructed by expert modeling using the APDTKTQ sequence [33,46].

All structures were equilibrated and relaxed during 100 ns of MD production run in water with NaCl concentration of 115 mM to obtain equilibrium conformations.

### 4.2. Docking Procedure

Equilibrium structures of Aβ_40_ peptide, Aβ_42_ peptide, the isoD7-Aβ_42_ and pS8-Aβ_42_ modifications, RP-1 and RAP peptides were submitted to global full-blind docking with RAGE protein as a receptor with servers LzerD [52], ATTRACT [53], PatchDock [54], ClusPro [55], HDOCK [56] and ZDOCK [57] and to the targeted docking with the PatchDock [54] server. Isomerized and phosphorylated residues are not supported by the docking software, so equilibrium structures of isoD7-Aβ_42_ and pS8-Aβ_42_ with standard residues were used in docking, and subsequently were mutated into iso-Asp7 and phospho-Ser8 after docking. For the FPS-ZM1 docking, Quick-Vina-W software was used [58]. All docking results were analyzed with the in-house software QASDOM (http://qasdom.eimb.ru/qasdom.html (accessed on 27 January 2022)) [59]. QASDOM was used to identify RAGE:Aβ_42_ interactions in the obtained complexes. The first 30 complexes from each server were used. A sum of all atomic contacts for each residue over all of the complexes was calculated by QASDOM; later, this parameter was used as “number of contacts”. Global docking was used for primary identification of the interaction sites with Aβ_40_, Aβ_42_, RP-1 and RAP peptides in the RAGE protein. The targeted flexible docking to the identified sites was used to obtain the best rated RAGE:Aβ_42_ isoforms, RAGE:Aβ_40_, RAGE:RAP and RAGE:RP-1 complexes with high affinity for subsequent MD simulations.

### 4.3. MD Simulations

The 100 ns MD simulations of each best-rated RAGE:Aβ_42_ isoform docking complex and RAGE:Aβ_40_ complex were performed. To ascertain stability of RAGE:Aβ_42_ isoform complexes in early and mature endosomes, they were modeled at pH 6.0 for 100 ns and at pH 5.5 for 100–250 ns. The following parameters were applied: for late endosomes, pH 5.5, concentration of chlorine 60 mM, sodium 20 mM, potassium 40 mM; and for early endosomes, pH 6.0, Cl and Na concentrations of 25 mM. In all other MD systems, the pH was 7.0 and Cl and Na concentrations were 0.115 mM. The protonation states were calculated and the systems were prepared using the Poisson–Boltzmann PDB2PQR server [60] (https://server.poissonboltzmann.org/ (accessed on 11 February 2022). The total charge of the complex at pH 5.5 was +6 e and at pH 6.0 was +5 e, while at neutral pH it was −1 e. The 100 ns MD simulations of the best rated RAGE:RAP, RAGE:RP-1 and RAGE:FPS-ZM1 docking complexes were performed.

All molecular complexes were subject to energy minimization with the consecutively applied steepest descent and conjugated gradients algorithms before MD modeling. Then, they were equilibrated in water and saline solution under position restraints for 1 ns in the NVT and NPT ensembles, respectively. In all models, the CHARMM36 forcefield [61] was used except for the systems with RAP and RP-1 peptides. For RAP and RP-1 modeling, the AMBER forcefield [62] was used to accommodate protected ends not supported by the CHARMM36 forcefield. MD calculations were performed with the particle mesh Ewald technique with the repeating boundary conditions and 1 nm cut-offs. The LINCS constraint algorithm with a 2-fs time step was applied. A constant temperature of 300 K was maintained throughout computations with two coupling groups.

The umbrella sampling [63] was performed for the RAGE:Aβ_42_, RAGE:isoD7-Aβ_42_ and RAGE:pS8-Aβ_42_ complexes after 50 ns of MD to obtain a potential of the mean force curve and to calculate the ∆G value for the binding/unbinding process. For the umbrella sampling of the RAGE:Aβ_42_ complex, 36 windows were chosen. These windows spanned the RAGE:Aβ_42_ center of mass (COM) separations from 1.2 nm to 6.2 nm. For the umbrella sampling of the RAGE:isoD7-Aβ42 complex, 33 windows were chosen. These windows spanned the RAGE:Aβ_42_ COM separations from 0.8 nm to 7.1 nm. For the umbrella sampling of the RAGE:pS8-Aβ_42_ complex, 38 windows were chosen. These windows spanned the RAGE:Aβ_42_ COM separations from 1.9 nm to 7.5 nm. The spacings between each window were 0.1 nm for the first 2 nm, 0.2 nm for next 2 nm and subsequently 0.3 nm, which enabled sufficient sampling. When required, additional windows were launched. The pulling was accomplished by applying a harmonic force with a force constant of 1000 kJ mol^−1^ nm^−2^. For each window, a 10 ns MD simulation was performed. Free energy was calculated for each final configuration using the weighted histogram analysis method (WHAM) [64].

## 5. Conclusions

Computer modeling showed that all Aβ isoforms can be transported through the cell as part of a complex with RAGE since all of the studied Aβ isoforms (Aβ_42_, Aβ_40_, pS8-Aβ_42_, isoD7-Aβ_42_) form stable complexes with RAGE both at a normal and acidic pH. Docking data showed novel interfaces for RAGE interaction with Aβ that were not previously studied. The main interaction in RAGE occurs for Aβ_42_ at the region between the V and C1 domains and the neighboring residues, while in the case of Aβ_40_ this region is much more extended and incorporates residues not involved in the interface for Aβ_42_. For the best-rated docking complexes of RAGE with Aβ_42_, pS8-Aβ_42_ and isoD7-Aβ_42_ peptides, the free energy of binding corresponds to the picomolar binding constant and the Aβ_42_ peptide has a higher binding constant compared with pS8-Aβ_42_ and isoD7-Aβ_42_ peptides. According to our data, the most promising inhibitor of Aβ interaction with RAGE is RP-1 as it closely overlaps the interaction interface with Aβ.

## Data Availability

This study did not report any data.

## Supplementary Materials

The following supporting information can be downloaded at: https://www.mdpi.com/article/10.3390/ijms231911816/s1.
