# Supplementary material for: Docking and Molecular Dynamics-Based Identification of Interaction between Various Beta-Amyloid Isoforms and RAGE Receptor
Source: Int J Mol Sci. 2022 Oct 5;23(19):11816. doi: 10.3390/ijms231911816 (PMC9570301; doi:10.3390/ijms231911816)
Supplement: Supplementary file 1 [file ijms-23-11816-s001.zip › ijms-1839760-supplementary.pdf]

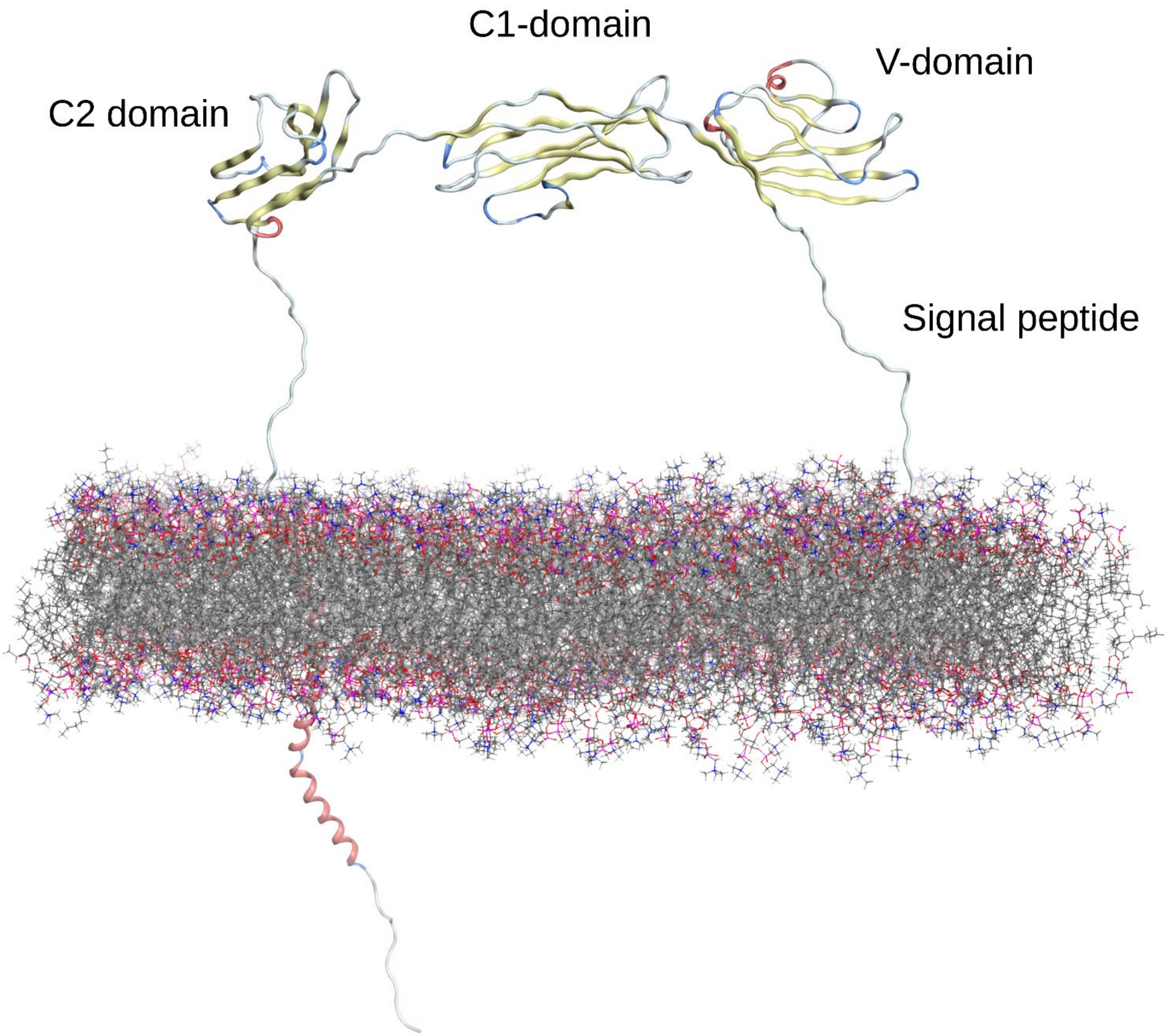

Figure S1. Equilibrium RAGE protein structure after 50 ns of MD with DDPC membrane. α-helices are colored red, β-sheets are colored yellow, H-bonded turns are colored blue.

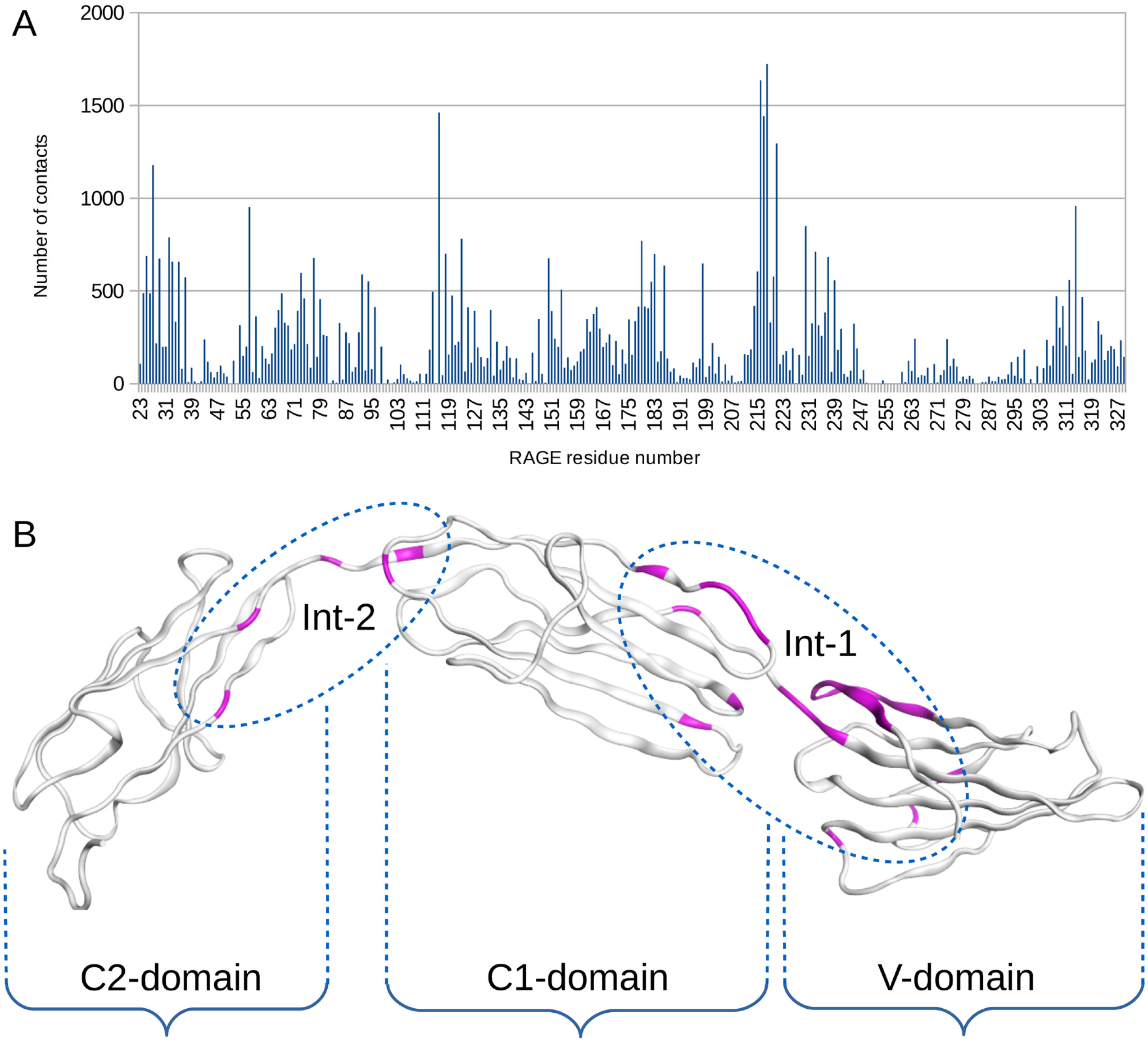

Figure S2. Global docking results of Aβ$_{42}$ to RAGE protein performed on servers LzerD, ATTRACT, PatchDock, ClusPro, Hdock and Zdock and analyzed with QASDOM software. 30 best complexes from each server were used for analysis, except 10 complexes from ZDock. **A** – Contact frequency histogram for RAGE residues over all 160 complexes. **B** – RAGE interaction interface with Aβ$_{42}$. Residues with more than 600 contacts are highlighted with magenta. N-terminus is at the right and C-terminus is at the left. The interaction areas are shown with ellipses.

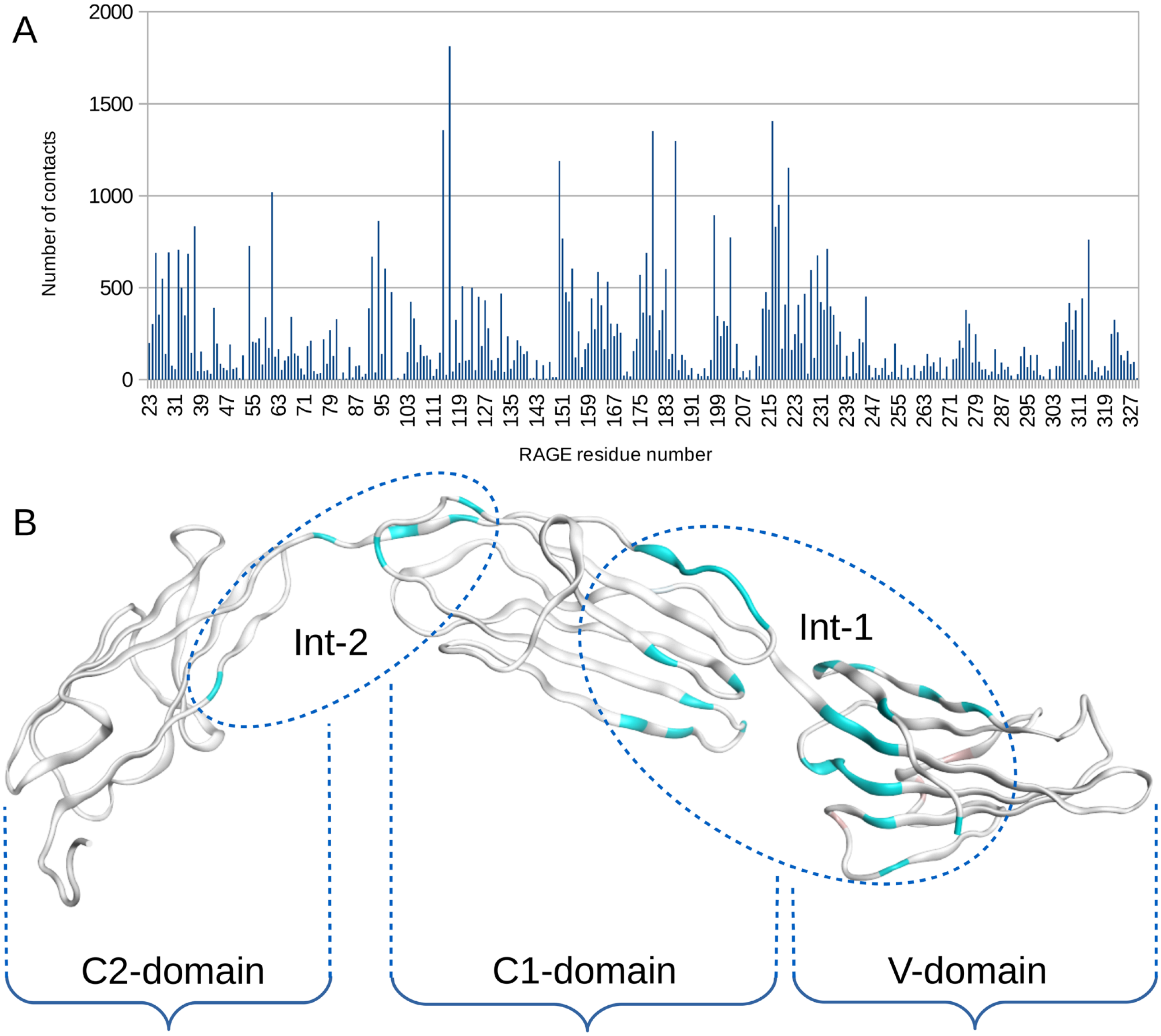

Figure S3. Global docking results of Aβ$_{40}$ to RAGE protein performed on servers LzerD, ATTRACT, PatchDock, ClusPro, Hdock and Zdock and analyzed with QASDOM software. 30 best complexes from each server were used for analysis, except 10 complexes from ZDock. **A** – Contact frequency histogram for RAGE residues over all 160 complexes. **B** – RAGE interaction interface with Aβ$_{40}$. Residues with more than 600 contacts are highlighted with cyan. N-terminus is at the right and C-terminus is at the left. The interaction areas are shown with ellipses.

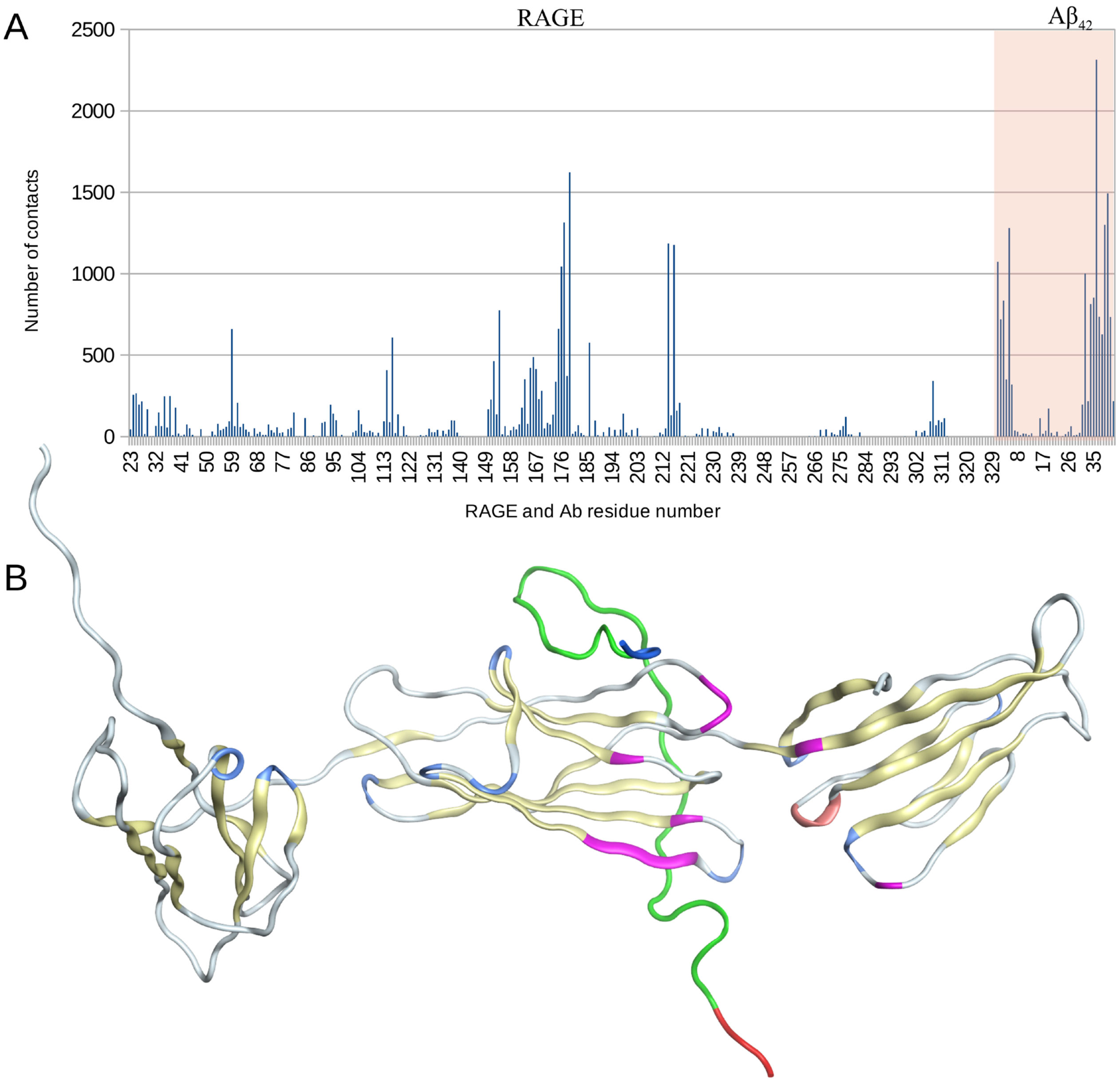

Figure S4. Global docking results of Aβ42 peptide to RAGE complex with another Aβ42 molecule. The initial complex was obtained by docking and 50 ns MD. **A** – Contact frequency histogram for RAGE residues over all 160 complexes. The residues of Aβ42 molecule bound to RAGE are highlighted with beige on the histogram. **B** – RAGE interaction interface with the docked Aβ42. Residues with more than 600 contacts are highlighted with magenta. N-terminus is at the right and C-terminus is at the left. Aβ42 is colored green, N-terminus is colored with blue and C-terminus with red. The RAGE secondary structure is marked with color: red for helices, yellow for sheets, blue for turns.

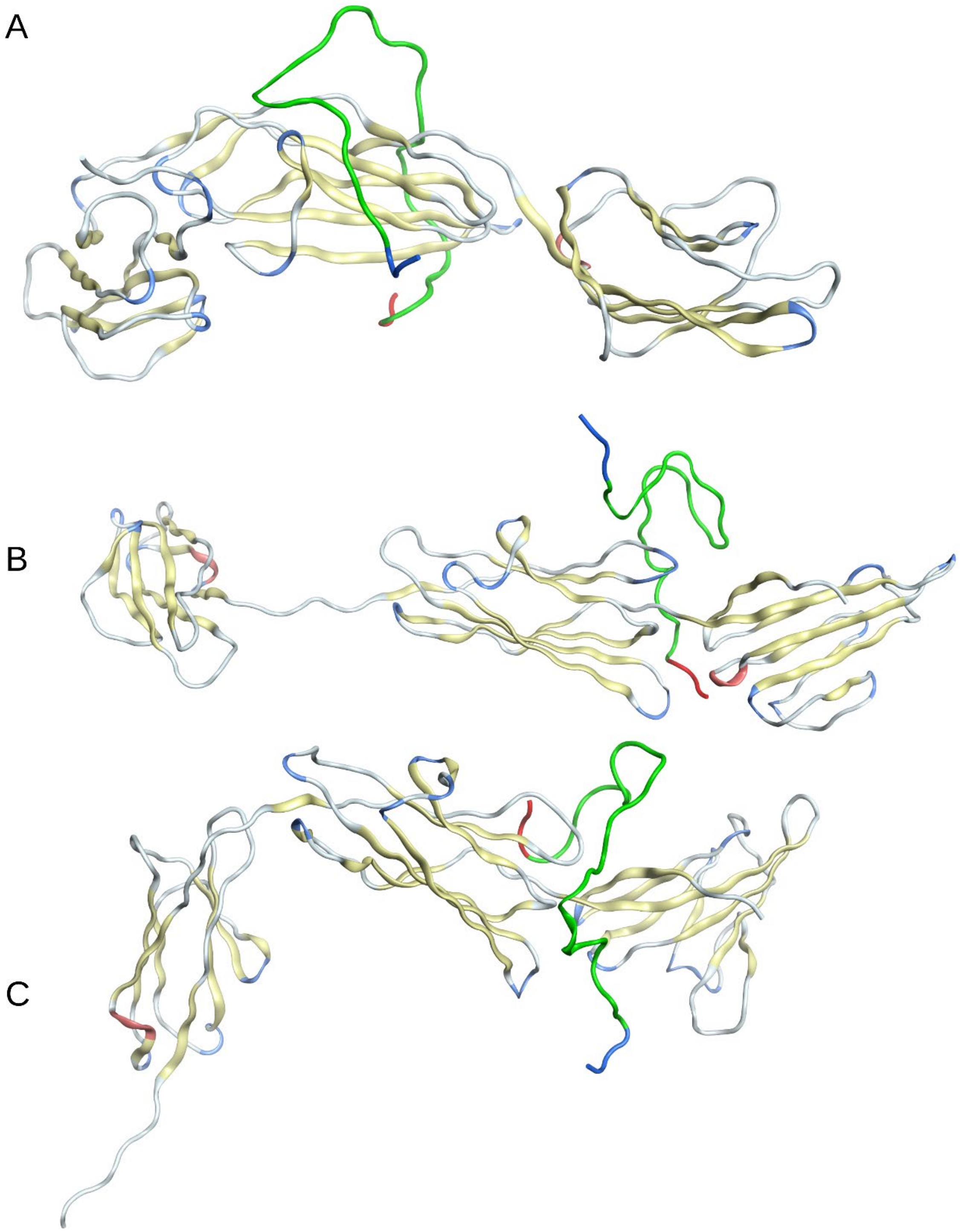

Figure S5. Best docking RAGE complexes with three Aβ$_{42}$ isoforms after 100 ns of MD at acid pH. **A** – RAGE with Aβ$_{42}$ at pH 6.0, **B** – RAGE withpS8-Aβ$_{42}$ at pH 5.5, C- RAGE with isoD7-Aβ$_{42}$ at pH 5.5. The Aβ$_{42}$ peptides are colored with green, N-termini are highlighted with blue, C-termini are highlighted with red. The secondary structure on RAGE is highlighted with color: red for helices, yellow for sheets, blue for turns.

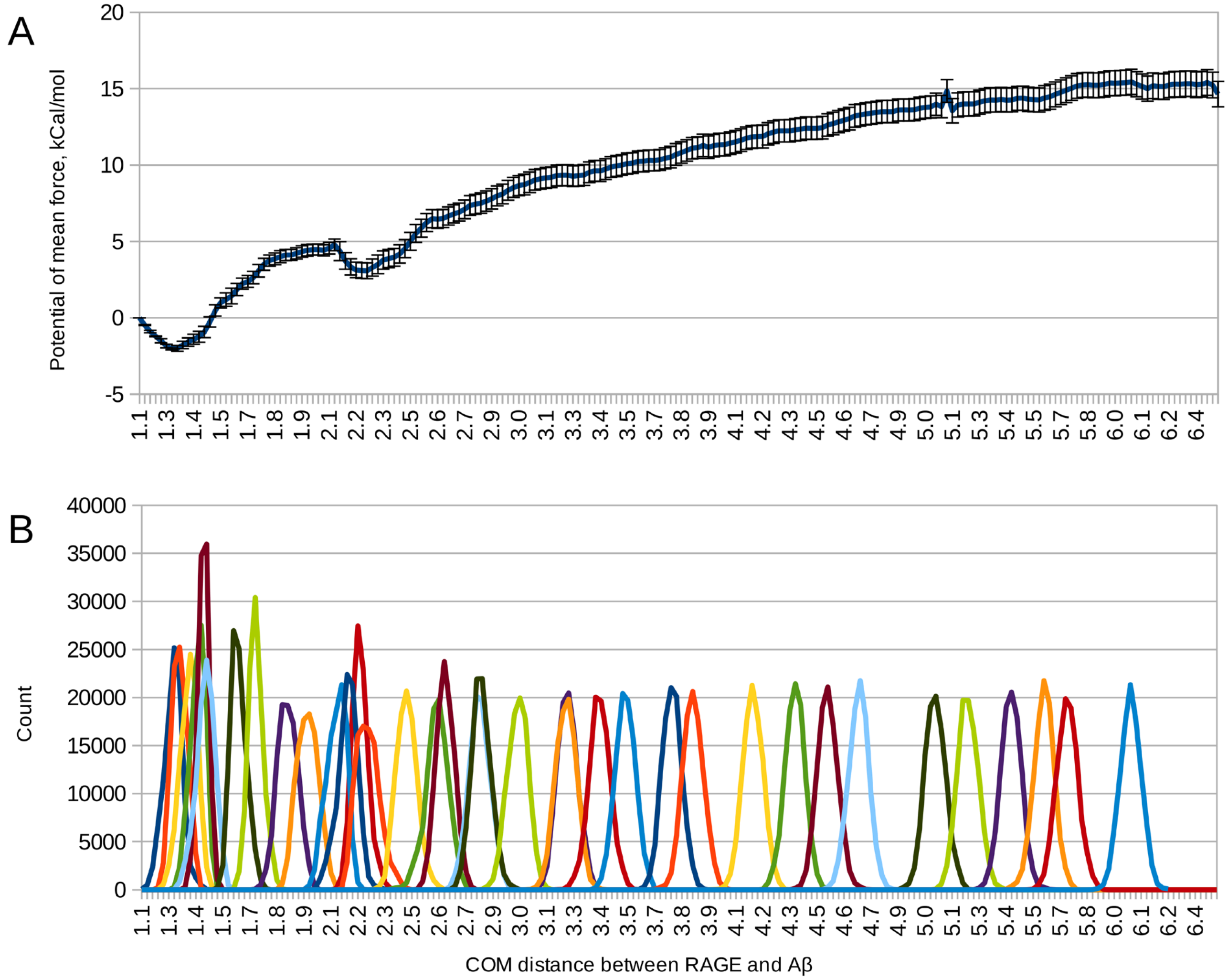

Figure S6. A – Potential of the mean force (PMF) of the Aβ_{42} peptide, as it moves from the RAGE protein into water. **B** – structure distribution over all simulation windows after 10 ns of MD. Each umbrella histogram representing a simulation window is shown with different color.

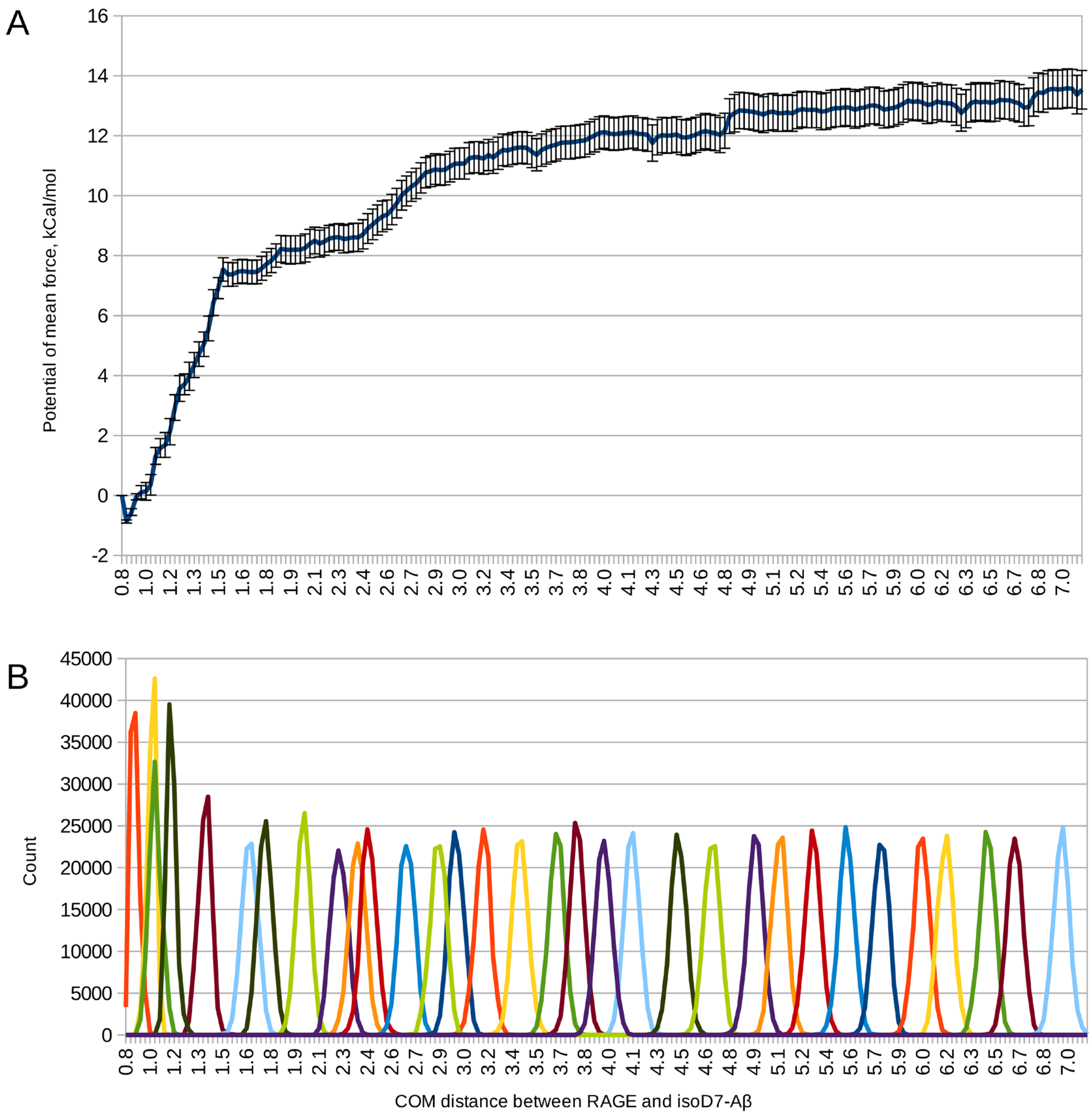

Figure S7. A – Potential of the mean force (PMF) of the isoD7-Aβ_{42} peptide, as it moves from the RAGE protein into water. **B** – structure distribution over all simulation windows after 10 ns of MD. Each umbrella histogram representing a simulation window is shown with different color.

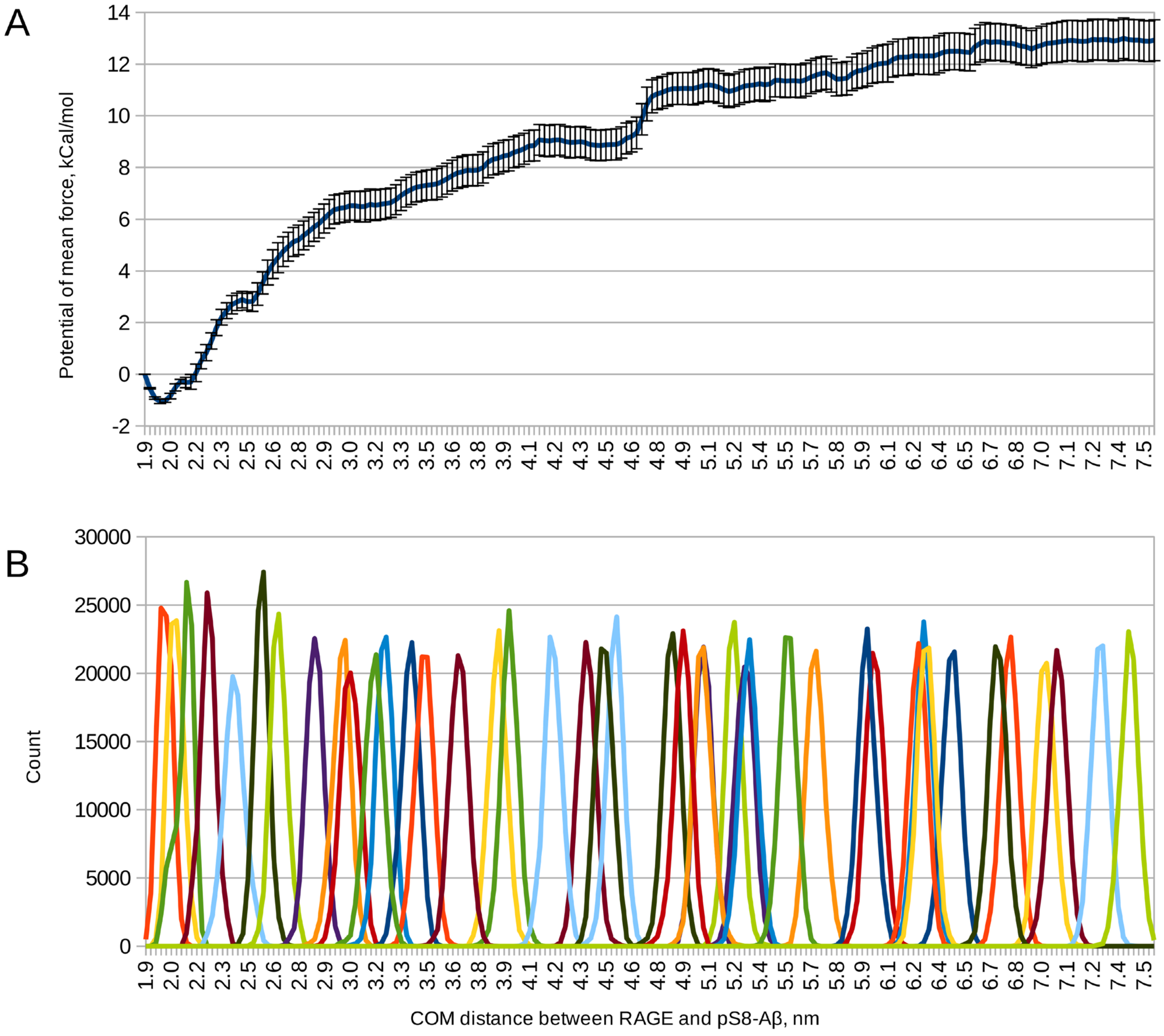

Figure S8. A – Potential of the mean force (PMF) of the pS8-Aβ_{42} peptide, as it moves from the RAGE protein into water. **B** – structure distribution over all simulation windows after 10 ns of MD. Each umbrella histogram representing a simulation window is shown with different color.

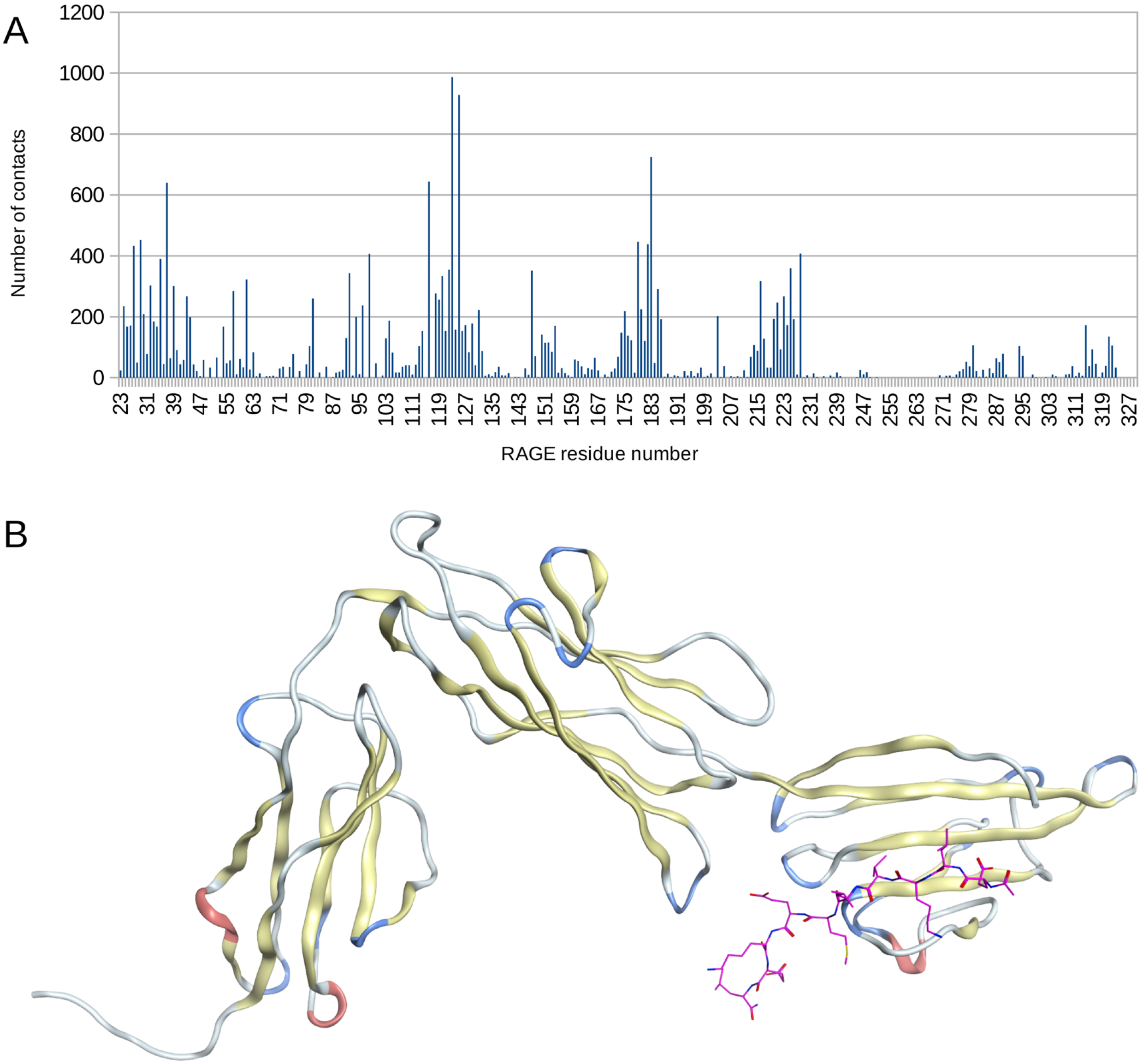

Figure S9. Global docking results of RAP peptide to RAGE protein performed on servers LzerD, ATTRACT, PatchDock and analyzed with QASDOM software. **A** – Contact frequency histogram for RAGE residues over all docking complexes. **B** – final complex structure after 100 ns of MD. The secondary structure on RAGE is highlighted with color: red for helices, yellow for sheets, blue for turns.

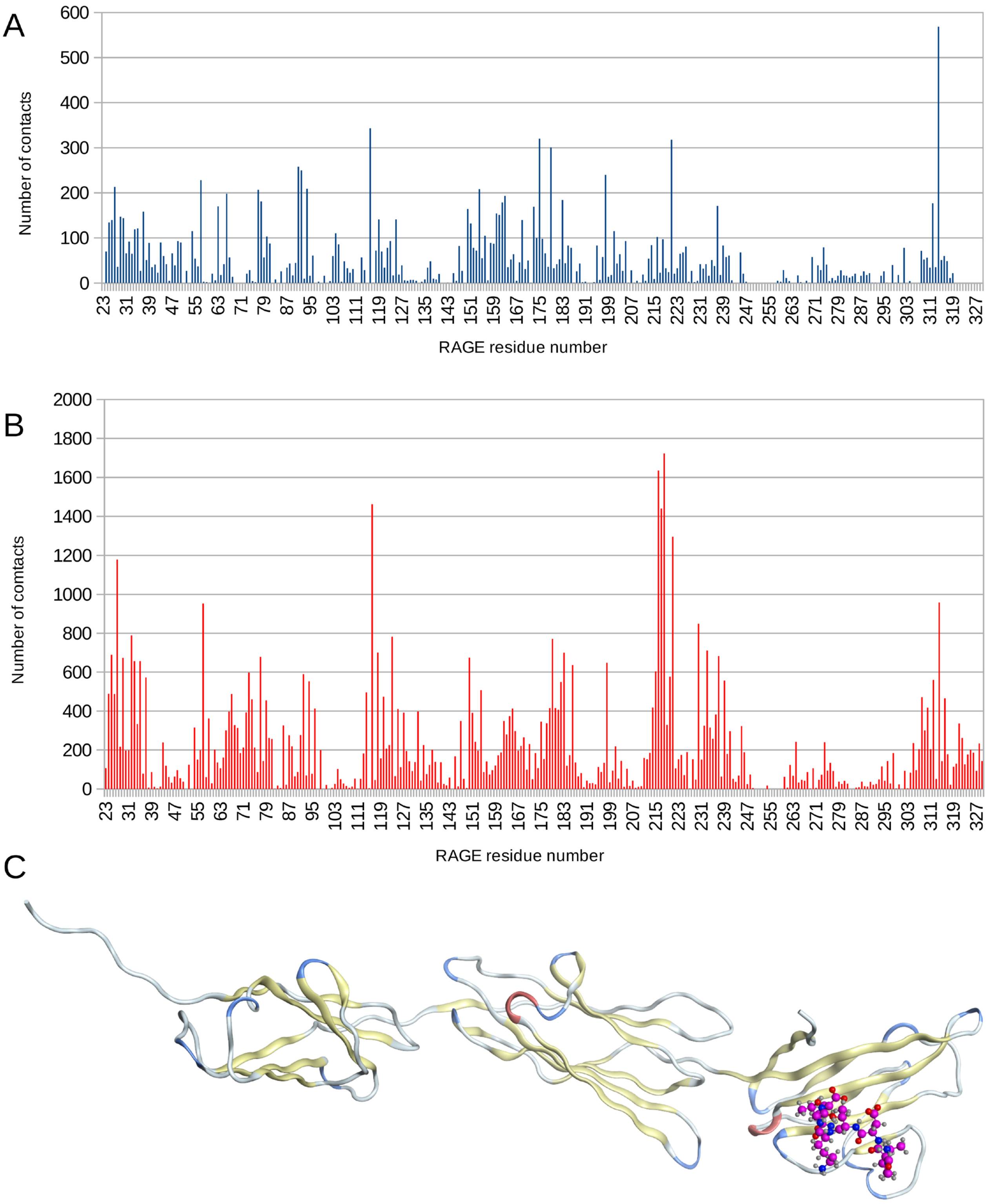

Figure S10. Global docking results of RP-1 peptide to RAGE protein performed on servers LzerD, ATTRACT, PatchDock and analyzed with QASDOM software. **A** – Contact frequency histogram for RAGE residues over all docking complexes. **B** – Contact frequency histogram for RAGE residues in complex with $A\beta_{42}$ peptide, **C** – final RAGE:RP-1 structure after 100 ns

of MD. The secondary structure on RAGE is highlighted with color: red for helices, yellow for sheets, blue for turns.

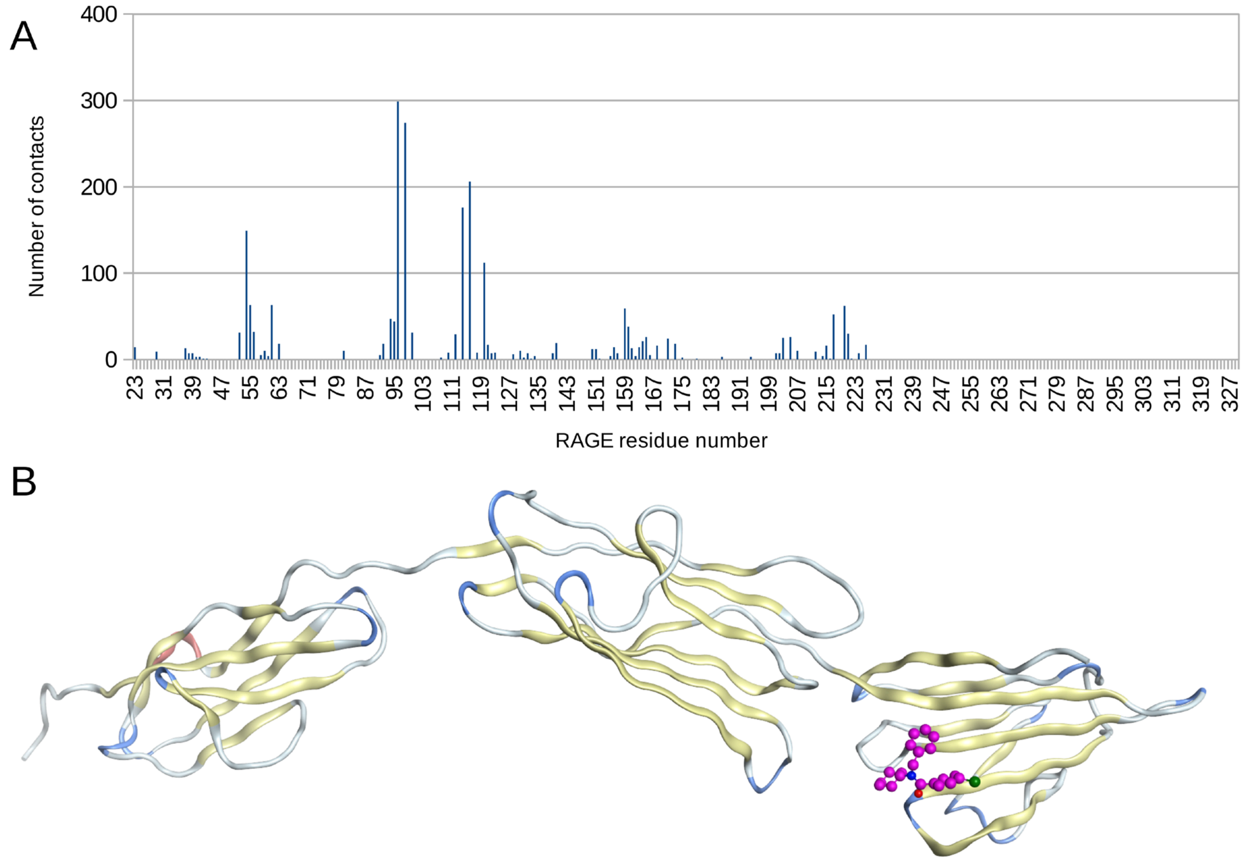

Figure S11. Global docking results of FPS-ZM1 to RAGE protein performed by Quick-Vina-W software and analyzed with QASDOM software. 30 best complexes were used for analysis. **A** – Contact frequency histogram for RAGE residues, **B** – structure of best rated complex after 100 ns of MD simulation. The secondary structure on RAGE is highlighted with color: red for helices, yellow for sheets, blue for turns.
